# Methomyl, imbraclaobrid and clethodim induced cytomixis and syncytes behaviors in PMCs of *Pisum sativum* L: Causes and outcomes

**DOI:** 10.1016/j.sjbs.2022.103390

**Published:** 2022-07-21

**Authors:** Sazada Siddiqui, Sulaiman A. Alrumman

**Affiliations:** Department of Biology, College of Science, King Khalid University, Abha 61413, Saudi Arabia

**Keywords:** Cytomixis, Syncytes, Pollen fertility, Pesticides, Methomyl, Imbraclaobrid, Clethodim, *Pisum sativum* L

## Abstract

Cytomixis is a common phenomenon observed in meiotic cells such as anther which is influenced by various factors. Use of pesticides is a common practice in agriculture. However, it is not known whether pesticides can induce cytomixis in plant cells and induce genetic variation. To understand this, the present study was planned to assess the cytomixis and syncytes behaviors in PMCs of *Pisum sativum* L. Seeds of *P. sativum* (Family: Fabaceae) were treated with different concentrations of commonly used pesticides methomyl (ME), imbraclaobrid (IM) and clethodim (CL). Seeds were treated with various concentrations (0.1, 0.2, 0.3, 0.4 and 0.5% of ME, IM and CL prepared in water) for 1 and 3 h. Effect of pesticides on pollen fertility, frequency of cytomixis, and kind of cytomixis cells was assessed. In the cytomixis cells, the cytomictic channel (CC) and direct fusion (DF), and various stages of meiosis (PI, MI, AI and TI) with cytomixis cells were observed. In addition, frequency of syncytes cell and their various stages of meiosis I (PI, MI, AI and TI) in pollen mother cells (PMCs) was assessed. During the microsporogenesis in *P. sativum,* the occurrence of cytomixis and syncytes at various stages of meiosis I were seen. The formation of cytoplasmic channels and direct fusing of pollen mother cells (PMCs) were both seen to cause cytomixis, with the former being more common than the latter. The percentage of PMCs with cytomixis and syncytes cells increased with increase in the concentration of pesticides. The result of the present investigation indicates that commonly used pesticides ME, IM, and CL have a significant effect on pollen fertility, frequency of cytomixis, and kind of cytomixis cells, the cytomictic channel (CC) and direct fusion (DF), in addition, frequency of syncytes cell and their various stages of meiosis I (PI, MI, AI and TI) in pollen mother cells (PMCs) on *P*. *sativum.*

## 1 Introduction

The movement of nuclei or fragments amongst plant cells is called cytomixis. This occurrence is most often observed in male meiosis and has been reported in microsporogenesis of more than 400 higher plant species so far (Girjesh and Shefali, 2020, Paez et al., 2021, Mursalimov et al., 2022). Cytomixis has sparked interest due to the unspecified mechanisms that allow nucleus to cross the cell wall. Though cytomixis has been observed in a variety of plants, its significance in plant physiology is still poorly understood. It is presumed to have evolutionary importance, since the transmission of genetic material amongst meiocytes can alter the karyotype of pollen produced. Earlier studies suggest that cytomixis is influenced by (i) genetic factors (Kaul and Nirmala, 1991); (ii) irregular cell wall development in premeiotic divides (Kamra, 1960); (iii) exposure to chemical agents (Amer and Mikhael, 1986, Sinha, 1988); (iv) modifications in the microenvironment of damaged anthers (Koul, 1990); (v) exposure to gamma radiation (Amma et al., 1990); and (vi) environmental pollutants (Haroun et al., 2004, Harshita and Girjesh, 2018). Cytomixis has been observed in various plant cells like tapetal (Cooper, 1952), root meristem (Tarkowska, 1960), graminaceous plant’s proembryos (Klyuchareva, 1983), ovarian (Koul, 1990), anthers vegetative tissue (Wang et al., 2004), tree shoot apex (Guzicka and Wozny, 2005) and woody plant’s apical meristem.

Cytomixis is divided into three different groups on the basis of intensity: mild (local), severe, and damaging (Mursalimov et al., 2013). According to Kravets (2012), local cytomixis has no detrimental impact on meiosis in contrast to the severe and damaging kind of cytomixis, which produces multiple cell autolysis as well as meiotic instabilities. The aberrations caused by cytomixis include the formation of PMCs with syncytes, leading to the sterility of pollen grains. These aberrations may reduce fertility (Singhal and Kumar, 2008). Due to the creation of syncytes, that results in the formation of 2n gametes, cytomixis is given a specific attention in biological research. Formation of syncytes is due to the union of two or more nuclei or PMCs, usually during preliminary prophase of I^st^ meiotic division, resulting in the production of 2n gametes by syncytes following meiosis (Sarbhoy, 1980). Cytomixis is generally recognized as a mutant, hybrid, and aneuploid attribute (Mursalimov and Deineko, 2018).

Various exogenous factors have shown to induce cytomixis in plants. The most common factors contributing to cytomixis are environmental stresses and pollution (Malallah and Attia, 2003, Kumar and Tripathi, 2008) and temperature effects (Basavaiah and Murthy, 1987). Use of pesticides has become a routine practice in agriculture in recent years in order to achieve effective pest control and increase the crop yield. Organophosphorous pesticides like ME, IM and CL are commonly used in KSA. It is evident that these pesticides can induce several cytological changes in the plants (Lukaszewicz et al., 2019, Siddiqui and Alrumman, 2020a, Siddiqui and Alrumman, 2020b, Tudi et al., 2021, Meshram et al., 2022). Spraying seeds with pesticides is a common practice in seed storage to prevent damage due to pest attack. However, there are no reports in the literature indicating whether they cause any cytomixis in plant cells. Therefore, the present investigation was planned to understand the effect of exposure of most commonly used pesticides such as ME, IM and CL when sprayed on seeds using *Pisum sativum* L as an experimental model. Further, to understand the association between cytomixis and pollen fertility and to investigate the effects of syncytes formation by cytomixis transmigration in *P. sativum*.

## 2 Materials and methods

### 2.1 Procurement of seeds and chemical

Healthy and fresh seeds of *P. sativum* were procured from a local market of Abha, Asir province, K.S.A. Pesticides methomyl (C_5_H_10_N_2_O_2_S) and imbraclaobrid (C_9_H_10_ClN_5_O_2_) were procured from Aleseba Est. for trading and contracting, Saudi Arabia. Clethodim (C17H26ClNO3S) was purchased from Saudi Delta Company.

### 2.2 Agroclimatic conditions of the experimental site

The present experiment was carried out during the Rabi season (October to November) in the experimental fields of the Department of Botany, College of Science, AL-Farra Campus, King Abdul Malik Road, King Khalid University, Abha, K.S.A.

Abha is situated in Asir's southern region of K.S.A. at GPS (18° 13′ 0.4692′' N and 42° 30′ 13.5540′' E) and at an altitude of around 2,270 m ASL. The area has a semi-arid climate that is affected by the city's high elevation. The weather is moderate all year, but it gets notably cooler during the “low-sun” season (December to February). Temperatures in Abha rarely exceed 35 °C throughout the year. The city receives annually an average of 278 mm of rainfall, most of which falls between February and April and some in July and August.

### 2.3 Treatment and sowing

The seeds of *P. sativum* were soaked for 12 h in distilled water before being treated with varying concentrations (0.1, 0.2, 0.3, 0.4 and 0.5% diluted in distilled water) of ME, IM, and CL for 1 and 3 h with intermittent shaking in a mechanical shaker (Stuart Reciprocating, Model SSL2, Thomas Scientific, United States). The concentration of pesticides used in this study was based on previous reports (Siddiqui et al., 2012). To remove the pesticides adhering to seed coat completely, the seeds were rinsed with running tap water for 10 min. For comparison, a set of seeds treated in the same manner as those with the experimental seeds but without the pesticide treatment, which were considered as control seeds. In Rabi season (2020–2021), 6 sets of treated seeds along with the control seeds were sown individually using a complete randomized block design (CRBD) having 3 replicates. Each treatment group had 300 seeds. In each plot measuring 6 × 6 m, 100 seeds were sown with a seed-to-seed distance of 25 cm and row to row distance of 40 cm. Fertilizers were not applied to any of the treatment groups.

### 2.4 Collection and fixation of buds

Young flower buds were collected after 60 days of sowing from 30 to 40 randomly picked plants and were fixed for 24 h in freshly prepared Carnoy's fixative (6 parts alcohol: 3 parts chloroform: 1 part acetic acid). The buds were then rinsed and kept at 4 °C in 70% alcohol for meiotic investigations.

### 2.5 Meiotic study

#### 2.5.1 Cytomixis and syncytes cell study

Healthy, young, and fresh flower buds were picked from the experimental plants and fixed for 24 h in 1:3 acetic acid saturated with absolute alcohol and passed through 70% (volume by volume) alcohol to analyze the effect of the selected pesticides on the meiotic cells (cytomixis and syncytes cells). The acetocarmine squash procedure was used for the meiotic cell preparations (Siddiqui and Alrumman, 2020b). Slides were made by squashing the anthers in an acetocarmine stain. Minimum of 250 cells were scored for each bud using light microscope (Leica DM750 P, Leica Microsystems, Switzerland) under oil immersion (1000 X magnification). Permanent slides were prepared in normal butanyl alcohol (NBA) series, mounted in Canada balsam, and dried at 45 °C.

#### 2.5.2 Analysis of pollen grains

For pollen grain studies, flower buds of equal age were obtained from treated and control group and fixed in 70% alcohol. Pollens were stained with 1% propionocarmine to determine pollen fertility and sterility. Pollen grains with similar shape and size, stained dark purple, and loaded with nuclei as well as cytoplasm were considered fertile, whereas pollens with unequal shape and size, devoid of both cytoplasm and nuclei, stained colorless and pale yellow were considered sterile. Pollen fertility was measured as percentage of viable pollens to total pollens (Marks, 1954).

$$\mathrm{Fertilepollengrain}\left(\%\right)=\frac{\mathrm{No}\;.of\;viable\;pollen\;grain}{\mathrm{Total}\;\mathrm{no}.\;of\;pollen\;grains}100$$

### 2.6 Data analysis

Statistical analysis was carried out using one way ANOVA test applying GPIS software 1.13 (GRAPHPAD, California, USA) to find out the significance of differences in variables. All the outcomes were articulated in mean ± standard error.

## 3 Results

### 3.1 Effects of ME, IM and CL on pollen fertility

**Fig. 1:**
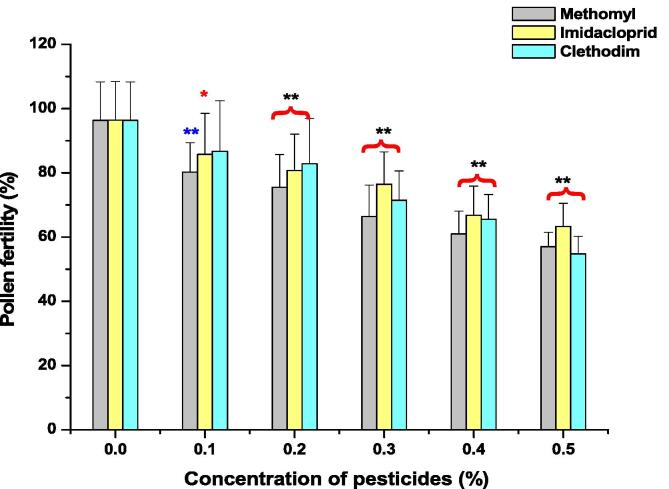
Effects of Methomyl, Imbraclaobrid and Clethodim on pollen fertility of *P. sativum* for 1 h. **p < 0.01 compared to control. *p < 0.05 compared to control. Data are mean of three replicates ± SE.

**Fig. 2:**
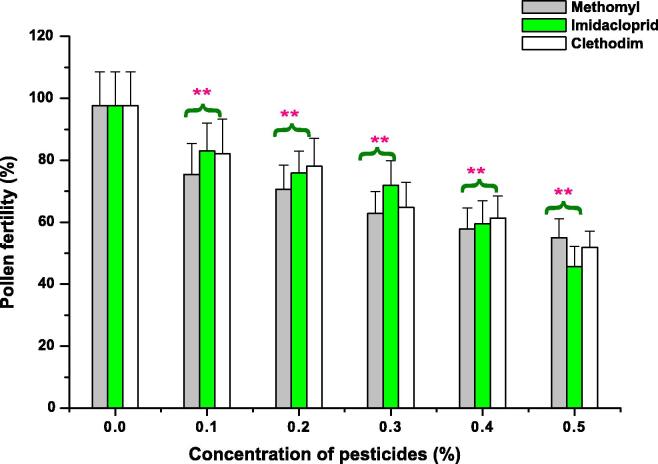
Effects of Methomyl, Imbraclaobrid and Clethodim on pollen fertility of *P. sativum* for 3 h. **p < 0.01 compared to control. Data are mean of three replicates ± SE.

**Fig. 3:**
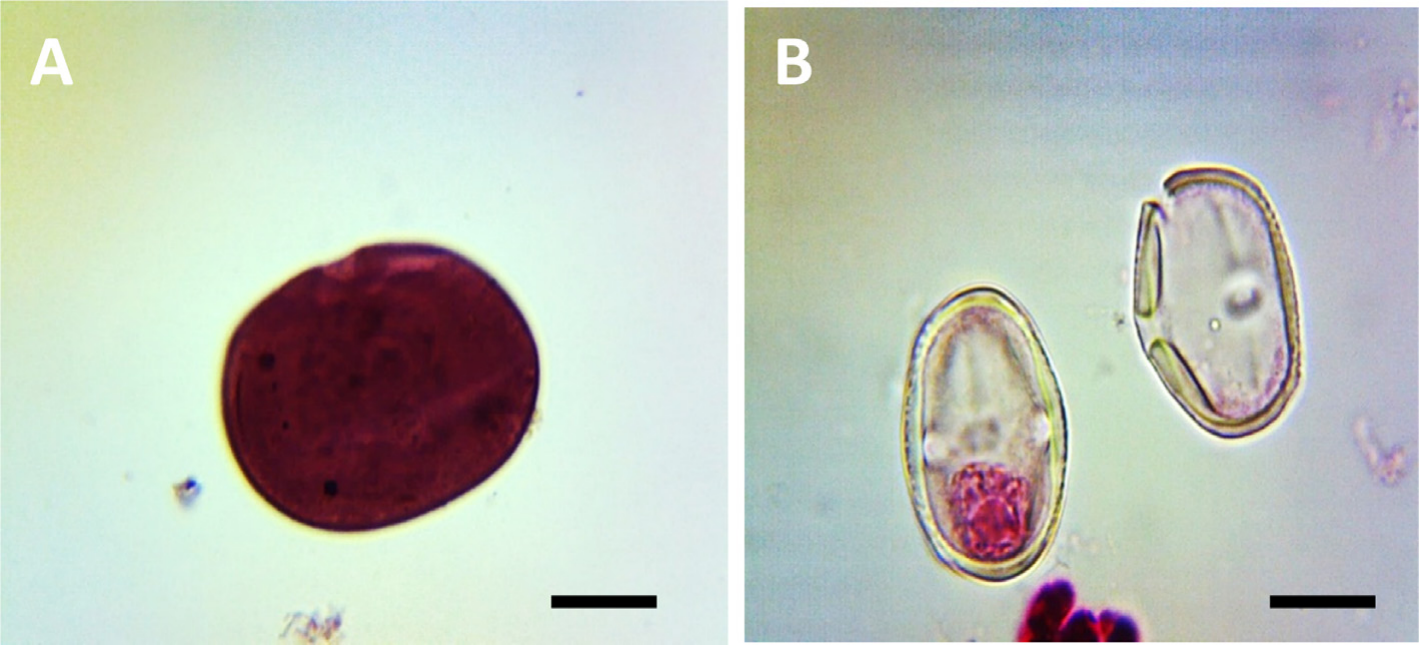
Pollen fertility induced by ME, IM and CL in PMCs of *P. sativum*, A. Fertile pollen grain in PMCs; B. Sterile pollen grain in PMCs of *P. sativum*, Scale bars = 10 μm.

As shown in Fig. 1, Fig. 2, Fig. 3 (A and B), percentage of PMCs exhibiting pollen fertility decreased with a rise in concentrations of ME, IM, and CL in a dose-dependent manner when exposed for 1 and 3 h. In control group, the pollen fertility was 96.34%. Significant decrease (p < 0.01) in percentage of pollen fertility was observed in seeds treated with 0.1 to 0.5% of ME, IM and CL for 1 and 3 h (except in 0.1% IM treated seeds which showed a significant decrease (p < 0.05) (85.75%) in percentage of pollen fertility and at 0.1% CL treated seeds, non-significant decrease (86.71%) in percentage of pollen fertility was reported when treated for 1 h. Minimum decline in pollen fertility was reported after 1 h treatment of ME (80.25%), IM (85.75%) and CL (86.17%) and after 3 h treatment of ME (75.45%), IM (82.98%), and CL (82.12%) at 0.1% which was very significant (p < 0.01) in comparison to control.

Pollen fertility was lowest in ME, IM and CL at 0.5% concentration when the seeds were exposed for 1 h (56.99%, 63.33% and 54.78% respectively). Similar trend was observed when the seeds were exposed for 3 h (54.99%, 45.65% and 51.89% in ME, IM and CL respectively). However, increasing the exposure duration did not seem to increase the pollen sterility in any of the pesticides.

### 3.2 Effects of ME, IM and CL on frequency of cytomixis in PMCs

No cytomixis cells were observed in control group (Table 1, Table 2). However, significant increase (p < 0.01) in percentage of cytomixis cells were observed in seeds treated with 0.1 to 0.5% of ME, IM, and CL for 1 and 3 h. Lowest number of cytomixis cells were reported in seeds exposed to 0.1% ME (40.12%), IM (43.98%) and CL (47.34%) whereas highest number of cytomixis cells were reported at 0.5% concentration in ME (75.12%), IM (82.11%), and CL (79.12%) when seeds were exposed for 1 h. Similarly, when seeds were exposed for 3 h with ME, IM and CL, lowest number of cytomixis cells were found in ME (55.23%), IM (48.11%) and CL (53.23%) at 0.1% concentration. Highest number of cytomixis cells were observed in ME (119.32%), IM (93.72%) and CL (85.32%) at 0.5% concentration. Overall, the effect was dose-dependent in nature for all the pesticides.

**Table 1:** The incidence of cytomixis in *P. sativum* in PMCs exposed to different concentrations of ME, IM and CL for 1 h.

Concentration (%)	No of cells in cytomixis	Types		No. of cells showing cytomixis at various stages of meiosis			
		DF	CC	PI	MI	AI	TI
0.0	0.00 ± 0.00	0.00 ± 0.00	0.00 ± 0.00	0.00 ± 0.00	0.00 ± 0.00	0.00 ± 0.00	0.00 ± 0.00
ME							
0.1	40.12 ± 3.44**	18.12 ± 3.27**	22.22 ± 3.25**	0.99 ± 0.08**	0.77 ± 0.03**	0.90 ± 0.04**	0.65 ± 0.03**
0.2	45.22 ± 4.33**	15.12 ± 2.23**	30.11 ± 5.12**	1.23 ± 0.70**	0.99 ± 0.04**	1.23 ± 0.07**	0.77 ± 0.04**
0.3	50.23 ± 3.45**	15.11 ± 2.23**	35.33 ± 6.12**	1.45 ± 0.91**	1.22 ± 0.04**	2.15 ± 0.90**	0.98 ± 0.04**
0.4	68.32 ± 4.21**	28.22 ± 4.13**	40.31 ± 4.12**	2.73 ± 1.01**	2.45 ± 0.78**	3.12 ± 1.00**	1.45 ± 0.06**
0.5	75.12 ± 5.99**	28.33 ± 7.45**	47.24 ± 5.32**	3.45 ± 1.34**	3.92 ± 0.90**	4.12 ± 1.20**	2.12 ± 0.60**
IM							
0.1	43.98 ± 10.12**	19.12 ± 6.33**	24.22 ± 9.88**	0.66 ± 0.02**	0.63 ± 0.02**	0.20 ± 0.001	0.68 ± 0.04
0.2	51.55 ± 8.11**	30.11 ± 8.80**	30.33 ± 8.77**	0.78 ± 0.03**	0.77 ± 0.06**	0.43 ± 0.03	0.91 ± 0.09*
0.3	54.67 ± 8.50**	35.34 ± 7.11**	35.22 ± 7.67**	0.88 ± 0.04**	0.99 ± 0.03**	1.21 ± 0.88**	1.23 ± 0.91**
0.4	74.98 ± 8.70**	42.01 ± 6.70**	42.23 ± 6.12**	0.99 ± 0.07**	1.23 ± 0.09**	2.11 ± 0.99**	2.54 ± 1.20**
0.5	82.11 ± 7.90**	50.12 ± 7.77**	50.11 ± 5.43**	1.23 ± 0.08**	1.53 ± 0.91**	2.11 ± 1.01**	3.15 ± 1.32**
CL							
0.1	47.34 ± 6.33**	22.23 ± 5.67**	25.44 ± 5.67**	0.34 ± 0.01	0.80 ± 0.01**	0.65 ± 0.02**	0.25 ± 0.03**
0.2	53.12 ± 5.77**	20.44 ± 6.63**	33.22 ± 5.43**	0.96 ± 0.05**	0.55 ± 0.05**	0.88 ± 0.05**	0.67 ± 0.04**
0.3	57.66 ± 8.99**	17.45 ± 4.33**	40.11 ± 7.40**	1.66 ± 0.33**	0.76 ± 0.06**	0.97 ± 0.07**	0.87 ± 0.23**
0.4	76.33 ± 9.33**	27.23 ± 6.70**	42.09 ± 6.32**	1.50 ± 0.79**	0.76 ± 0.03**	1.12 ± 0.30**	0.96 ± 0.31**
0.5	79.12 ± 11.33**	35.32 ± 9.66**	44.44 ± 8.44**	1.65 ± 0.76**	0.98 ± 0.09**	1.24 ± 0.40**	1.01 ± 0.09**

Data are mean of three replicates ± SE. 0.0 = Control, PI = Prophase I, MI = Metaphase I, AI = Anaphase I, TI = Telophase I; Total no. of PMCs Observed = 250.

** *p <* 0.01 compared to control

* *p <* 0.05 compared to control

**Table 2:** The incidence of cytomixis in *P. sativum* in PMCs exposed to different concentrations of ME, IM and CL for 3 h.

Concent ration (%).	No. of cells in cytomixis	Types		No. of cells showing cytomixis at various stages of meiosis			
		DF	CC	PI	MI	AI	TI
0.0	0.00 ± 0.00	0.00 ± 0.00	0.00 ± 0.00	0.00 ± 0.00	0.00 ± 0.00	0.00 ± 0.00	0.00 ± 0.00
ME							
0.1	55.23 ± 6.62**	25.11 ± 4.23**	30.12 ± 5.52**	0.99 ± 0.02**	0.65 ± 0.02**	1.25 ± 0.23**	0.82 ± 0.06**
0.2	72.11 ± 11.32**	30.22 ± 5.72 **	42.52 ± 7.82**	1.45 ± 0.24**	0.88 ± 0.05**	1.94 ± 0.64**	0.99 ± 0.04**
0.3	92.88 ± 8.82**	38.45 ± 6.12**	54.33 ± 9.23**	1.98 ± 0.08**	0.97 ± 0.07**	2.25 ± 0.54**	1.97 ± 0.064**
0.4	1.08.12 ± 16.1**	40.32 ± 5.32**	68.45 ± 10.12**	2.99 ± 0.90**	1.12 ± 0.24**	3.25 ± 0.98**	2.45 ± 0.59**
0.5	119.32 ± 19.2**	41.45 ± 5.32**	78.45 ± 12.48**	3.78 ± 1.10 **	2.73 ± 0.98**	4.98 ± 1.15 **	3.75 ± 0.98**
IM							
0.1	48.11 ± 7.62**	23.43 ± 4.79**	25.11 ± 4.32**	0.88 ± 0.04**	1.99 ± 0.98 **	0.50 ± 0.01**	0.99 ± 0.06**
0.2	58.52 ± 5.21**	22.23 ± 3.28**	36.25 ± 4.89**	0.98 ± 0.08**	1.01 ± 0.52**	0.90 ± 0.030**	1.25 ± 0.32**
0.3	64.32 ± 6.12**	29.45 ± 5.12**	35.43 ± 6.32**	1.00 ± 0.04**	1.35 ± 0.62**	1.00 ± 0.077**	2.15 ± 0.62**
0.4	81.51 ± 10.12**	34.15 ± 4.15**	47.44 ± 5.23**	1.52 ± 0.25**	1.45 ± 0.52**	2.15 ± 0.50**	3.15 ± 1.00**
0.5	93.72 ± 9.15**	40.22 ± 6.88**	53.32 ± 6.40**	2.17 ± 0.98**	2.73 ± 0.89**	2.99 ± 0.71**	4.15 ± 1.10**
CL							
0.1	53.23 ± 6.12**	30.77 ± 4.23**	3.52 ± 4.23**	0.63 ± 0.03**	0.34 ± 0.03**	0.15 ± 0.30**	0.76 ± 0.01**
0.2	59.44 ± 8.78**	39.67 ± 5.40**	20.12 ± 5.40**	0.65 ± 0.02**	0.76 ± 0.02**	0.26 ± 0.04**	0.78 ± 0.06**
0.3	64.53 ± 5.23**	40.55 ± 6.70**	25.52 ± 6.70**	1.53 ± 0.33**	0.76 ± 0.02**	0.46 ± 0.04**	0.87 ± 0.04**
0.4	79.55 ± 6.45**	50.33 ± 4.60**	29.77 ± 4.80**	1.20 ± 0.66**	0.96 ± 0.06**	0.66 ± 0.03**	1.55 ± 0.22**
0.5	85.32 ± 10.12**	47.44 ± 7.23**	38.78 ± 3.20**	1.30 ± 0.88**	1.01 ± 0.09**	0.91 ± 0.11**	1.80 ± 0.54**

Data are mean of three replicates ± SE. 0.0 = Control group, PI = Prophase I, MI = Metaphase I, AI = Anaphase I, TI = Telophase I; Total no. of PMCs Observed = 250.

*p < 0.05 compared to control

** *p <* 0.01 compared to control

### 3.3 Effects of ME, IM and CL on types of cytomixis cells such as direct fusion (DF) and cytomictic channel (CC) in PMCs

**Fig. 4:**
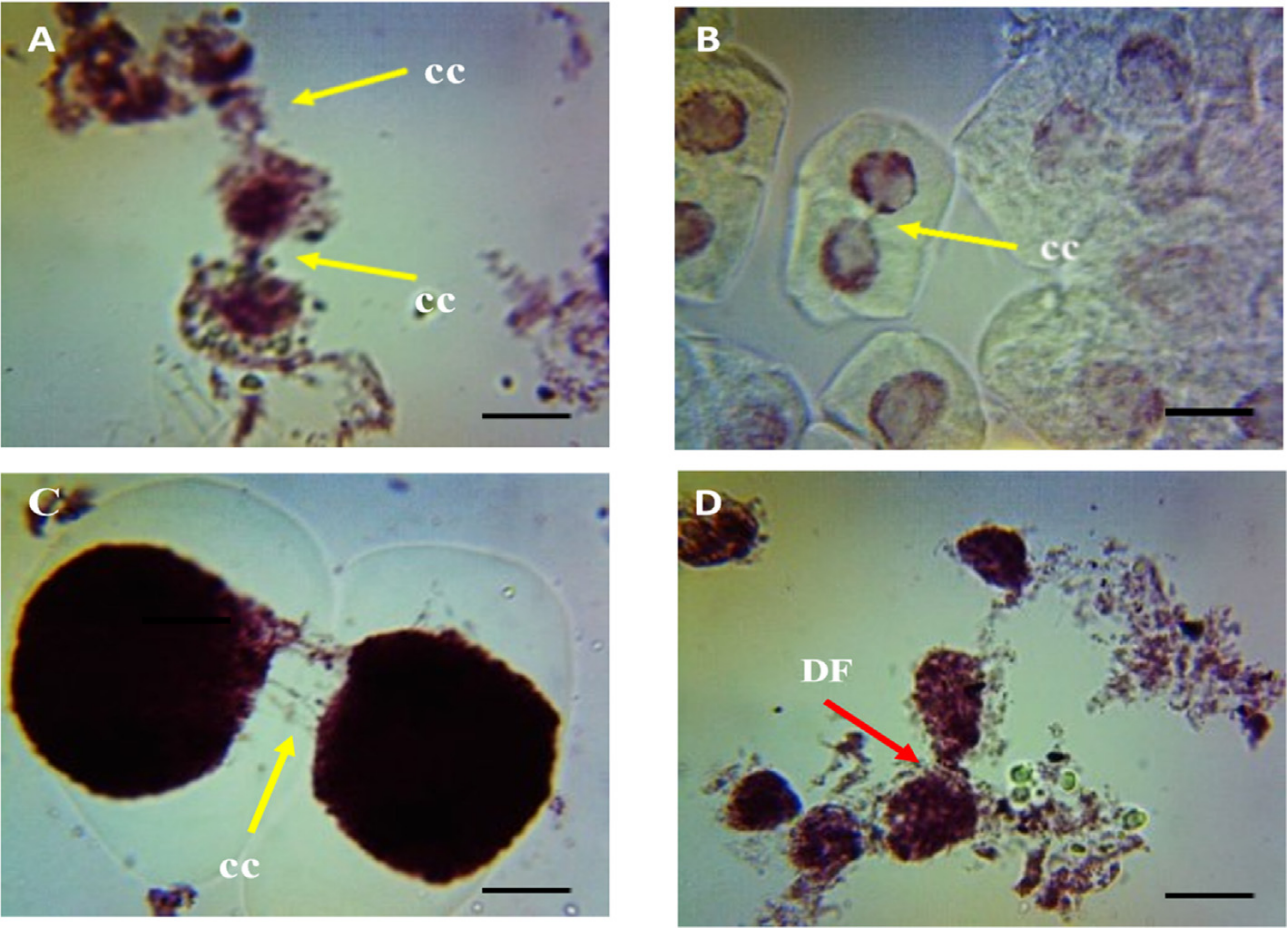
Cytoplasmic channel (CC) and direct fusion (DF) induced by ME, IM and CL in PMCs of *P. sativum*, (A, B and C) PMCs connected through a cytoplasmic channel, (D) PMCs connected through a direct fusion; Scale bars = 10 μm.

In control group there was no cytomixis cells showing direct fusion (DF) and cytomictic channel (CC) (Table 1, Table 2, Fig. 4 (A, B, C, and D). Significant increase (p < 0.01) in percentage of cytomixis cells with CC and DF were observed in seeds treated with 0.1 to 0.5% of ME, IM, and CL for 1 and 3 h. Minimum number of DF cells were reported at 0.1% in ME (18.12%), IM (19.12%) and CL (22.23%) whereas, maximum number of DF cells were reported at 0.5% concentration in ME (28.33%), IM (50.12%) and CL (35.32%) for 1 h treatment as compared with control. After 3 h treatment with ME, IM, and CL, the minimum number of DF cells were found at 0.1% concentration in ME (25.11%), IM (23.43%) and CL (23.52%) whereas maximum number of DF cells were observed in 0.5%, in ME (41.45%), IM (40.22%) and CL (38.78%) as compared with control.

Minimum number of CC cells were reported at 0.1% in ME (22.22%), IM (24.22%) and CL (25.44%) whereas its maximum number were reported at 0.5% concentration in ME (47.24%), IM (50.11%) and CL (44.44%) for 1 h treatment. After 3 h treatment with ME, IM and CL, CC cells were found at 0.1% concentration in ME (30.12%), IM (25.11%) and CL (30.71%) whereas maximum number of CC cells were observed in 0.5% in ME (78.45%), IM (53.32%) and CL (47.44%) as compared with control. Overall dose-dependent increase in DF and CC cells were reported in all concentrations in ME, IM, and CL in 1 and 3 h treatments.

### 3.4 Effect of ME, IM and CL on meiotic cells with cytomixis in PMCs

**Fig. 5:**
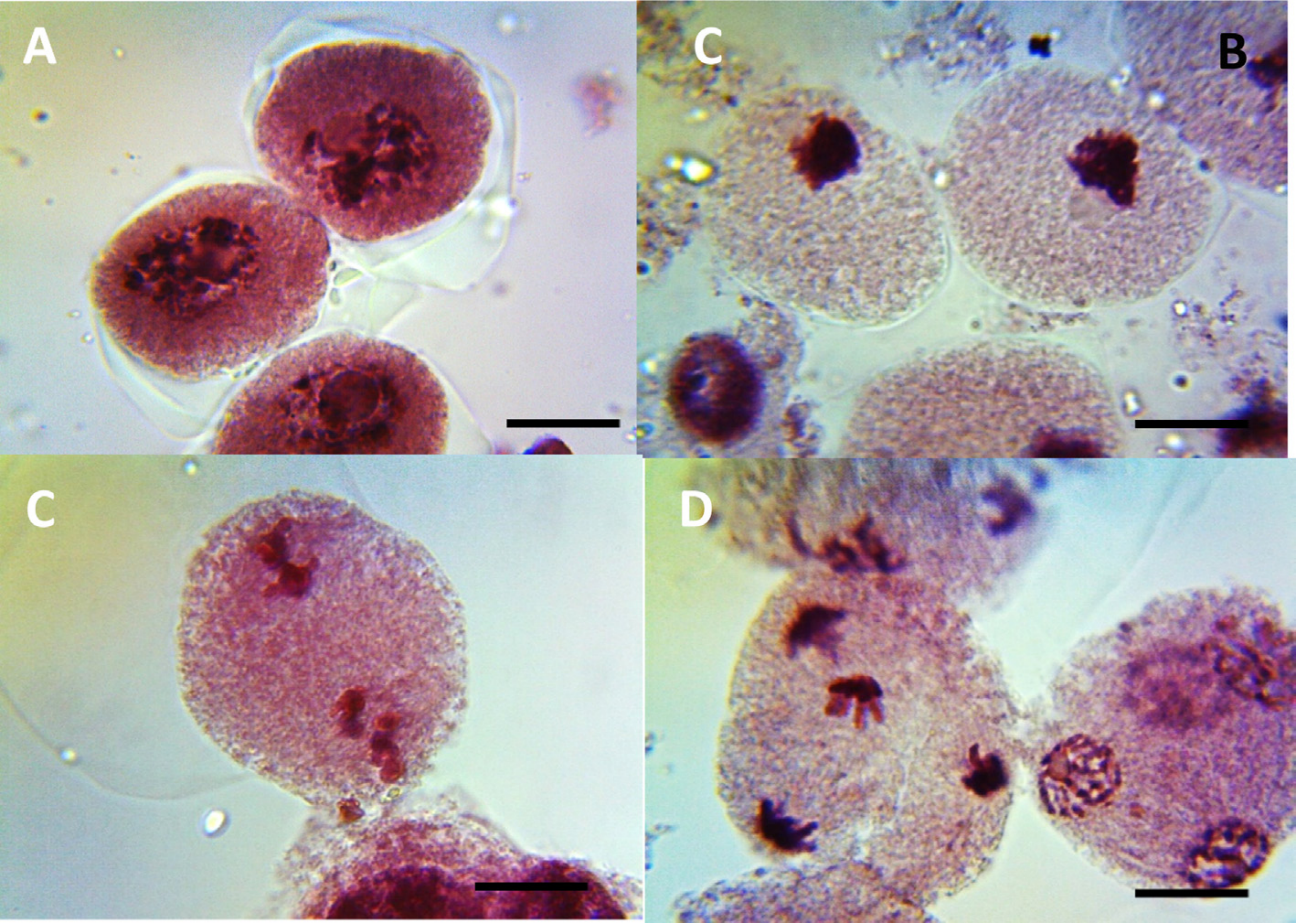
Cytomixis induced by ME, IM and CL in PMCs of *P. sativum*, A. Cytomixis in PI in PMCs, B. Cytomixis in MI in PMCs, C. Cytomixis in AI in PMCs, D. Cytomixis in TI in PMCs of *P. sativum,* PI = Prophase I; MI = Metaphase I; AI = Anaphase I; TI = Telophase I; Scale bars = 10 μm.

In control group, no cytomixis cells were observed in various stages of meiosis I in PMCs (PI, MI, AI and TI) (Table 1, Table 2 and Fig. 5 (A, B, C and D). In seeds exposed to ME for 1 h, significant increase (p < 0.01) in cytomixis cells in PMCs were observed in PI, MI, AI, and TI stage in all the concentrations.

In case of IB treated seeds for 1 h, significant increase (p < 0.01) in cytomixis cells in PI and MI stage of meiosis I in PMCs were observed when compared to control. However, in AI stage from 0.1 to 0.2% and in TI stage at 0.1% concentration, a non-significant increase in cytomixis cells in PMCs was observed. However, significant increase (in 0.1 and 0.2%, p < 0.05; 0.3–0.5%, p < 0.01) in cytomixis cells were observed at 1 h in comparison to control. In CL treated seeds at 0.1% concentration, non-significant increase in cytomixis cells were observed in PI stage of meiosis I in PMCs. However, at 0.2 to 0.5% concentration, significant increase (p < 0.01) in cytomixis cells were reported in comparison to control at 1 h. Further, in MI, AI, and TI stages of meiosis I at 1 h, significant increase (p < 0.01) in cytomixis cells was observed from 0.1 to 0.5% in comparison to control. Increasing the duration of exposure of all the pesticides to 3 h, resulted in a significant increase (p < 0.01) in cytomixis cells in PI, MI, AI, and TI stages of meiosis I in PMCs at all the concentrations in comparison to control.

### 3.5 Effects of ME, IM and CL on frequency of syncyte cells in PMCs

No syncyte cells were observed in control group (Table 3, Table 4). However, significant increase (p < 0.01) in percentage of syncyte cells were observed in seeds treated with 0.1 to 0.5% of ME, IM, and CL for 1 and 3 h treatment.

**Table 3:** The incidence of syncytes in *P. sativum* in PMCs exposed to different concentrations of ME, IM and CL for 1 h.

Concentration (%)	Frequency of syncytes	No. of cells showing syncytes at various stages of meiosis			
		PI	MI	AI	TI
0.0	0.00 ± 0.00	0.00 ± 0.00	0.00 ± 0.00	0.00 ± 0.00	0.00 ± 0.00
ME					
0.1	0.78 ± 0.05**	0.66 ± 0.02*	0.27 ± 0.01	0.33 ± 0.02	0.15 ± 0.01
0.2	1.23 ± 0.08**	1.45 ± 0.07**	0.69 ± 0.03*	0.74 ± 0.03*	0.55 ± 0.03*
0.3	1.59 ± 0.09**	2.12 ± 0.88**	0.99 ± 0.04**	0.94 ± 0.04**	1.98 ± 0.04**
0.4	2.61 ± 0.88**	2.75 ± 0.90**	1.23 ± 0.60**	1.02 ± 0.40**	2.23 ± 0.90**
0.5	2.78 ± 0.98**	3.12 ± 1.0**	1.56 ± 0.80**	1.95 ± 0.90**	3.13 ± 1.00**
IM					
0.1	0.91 ± 0.01**	0.44 ± 0.03**	0.30 ± 0.01	0.61 ± 0.03	0.51 ± 0.03**
0.2	1.25 ± 0.82**	0.97 ± 0.05**	0.83 ± 0.03**	1.87 ± 0.66**	0.98 ± 0.32**
0.3	2.75 ± 0.90**	1.0 ± 0.040**	1.33 ± 0.88**	2.11 ± 0.94**	1.10 ± 0.53**
0.4	3.24 ± 1.12**	1.21 ± 0.08**	1.50 ± 0.64**	2.78 ± 1.00**	1.16 ± 0.08**
0.5	4.94 ± 1.45**	1.45 ± 0.20**	2.14 ± 1.10**	3.12 ± 1.20**	1.60 ± 0.09**
CL					
0.1	2.00 ± 0.90**	0.12 ± 0.01	0.46 ± 0.05**	0.76 ± 0.06**	0.67 ± 0.03**
0.2	2.55 ± 0.54**	0.65 ± 0.06**	0.76 ± 0.15**	0.87 ± 0.04**	0.87 ± 0.04**
0.3	3.11 ± 0.70**	0.97 ± 0.05**	0.91 ± 0.10**	1.55 ± 0.32**	0.97 ± 0.05**
0.4	3.80 ± 0.90**	1.24 ± 0.20**	0.97 ± 0.23**	1.76 ± 0.77**	1.12 ± 0.32**
0.5	3.90 ± 0.97**	1.27 ± 0.41**	1.12 ± 0.70**	1.80 ± 0.63**	1.17 ± 0.56**

Data are mean of three replicates ± SE. 0.0 = Control, PI = Prophase I, MI = Metaphase I, AI = Anaphase I, TI = Telophase I; Total no. of PMCs Observed = 250.

** *p <* 0.01 compared to control

* *p <* 0.05 compared to control

**Table 4:** The incidence of syncytes in *P. sativum* in PMCs exposed to different concentration of ME, IM and CL for 3 h.

Concentration (%)	Frequency of syncytes	No. of cells showing syncytes at various stages of meiosis			
		PI	MI	AI	TI
00	00.0 ± 0.00	0.00 ± 0.00	0.00 ± 0.00	0.00 ± 0.00	0.00 ± 0.00
ME					
0.1	0.45 ± 0.02**	0.33 ± 0.25	0.48 ± 0.02	0.71 ± 0.04*	0.64 ± 0.05
0.2	0.76 ± 0.03**	1.55 ± 0.21**	0.86 ± 0.40**	0.98 ± 0.03**	0.92 ± 0.50**
0.3	0.99 ± 0.07**	1.75 ± 0.34**	1.12 ± 0.70**	1.23 ± 0.80**	1.99 ± 0.80**
0.4	1.99 ± 0.22**	2.12 ± 0.91**	2.32 ± 0.99**	2.45 ± 1.12**	2.23 ± 0.91**
0.5	2.45 ± 0.51**	3.98 ± 1.01**	3.25 ± 1.11**	3.12 ± 1.21**	3.42 ± 1.20**
IM					
0.1	1.25 ± 0.62**	0.77 ± 0.04**	0.77 ± 0.04**	0.98 ± 0.04**	0.97 ± 0.04**
0.2	2.25 ± 0.88**	1.32 ± 0.41**	0.99 ± 0.06**	1.99 ± 0.02**	1.45 ± 0.05**
0.3	2.75 ± 0.91**	1.17 ± 0.32**	1.47 ± 0.91**	2.32 ± 0.05**	1.98 ± 0.90**
0.4	3.15 ± 1.50**	2.12 ± 0.55**	2.15 ± 0.88**	2.78 ± 0.40**	2.34 ± 1.00**
0.5	3.32 ± 1.20**	2.89 ± 1.10**	3.45 ± 0.78**	3.77 ± 0.81**	2.78 ± 0.90**
CL					
0.1	1.53 ± 0.30**	0.25 ± 0.010**	0.40 ± 0.03**	0.46 ± 0.05**	0.34 ± 0.02**
0.2	1.59 ± 0.42**	0.55 ± 0.020**	0.45 ± 0.04**	0.66 ± 0.05**	0.96 ± 0.09**
0.3	2.59 ± 0.60**	0.77 ± 0.050**	0.61 ± 0.05**	1.10 ± 0.33**	1.23 ± 0.10**
0.4	3.72 ± 0.70**	0.96 ± 0.040**	0.98 ± 0.01**	1.55 ± 0.32**	1.17 ± 0.09**
0.5	4.86 ± 0.98**	1.10 ± 0.077**	1.25 ± 0.40**	1.70 ± 0.42**	1.27 ± 0.20**

Data are mean of three replicates ± SE. 0.0 = Control group, PI = Prophase I, MI = Metaphase I, AI = Anaphase I, TI = Telophase I; Total no. of PMCs Observed = 250.

** *p <* 0.01 compared to control

* *p <* 0.05 compared to control

Lowest number of syncyte cells were reported in seeds exposed to 0.1% ME (0.78%), IM (0.91%) and CL (2.0%) whereas highest number of syncyte cells were reported at 0.5% concentration in ME (2.78%), IM (4.94%) and CL (3.90%) when seeds were exposed for 1 h. Similarly, when seeds were exposed for 3 h with ME, IM, and CL, lowest number of syncyte cells were found in ME (0.45%), IM (1.25%) and CL (1.53%) at 0.1% concentration. Highest number of syncyte cells were observed in ME (2.45%), IM (3.32%), and CL (4.86%) at 0.5% concentration. Overall, the effect was dose-dependent in nature for all the pesticides.

### 3.6 Effects of ME, IM and CL on meiotic cells (PI, MI, AI and TI) with syncyte cells in PMCs

**Fig. 6:**
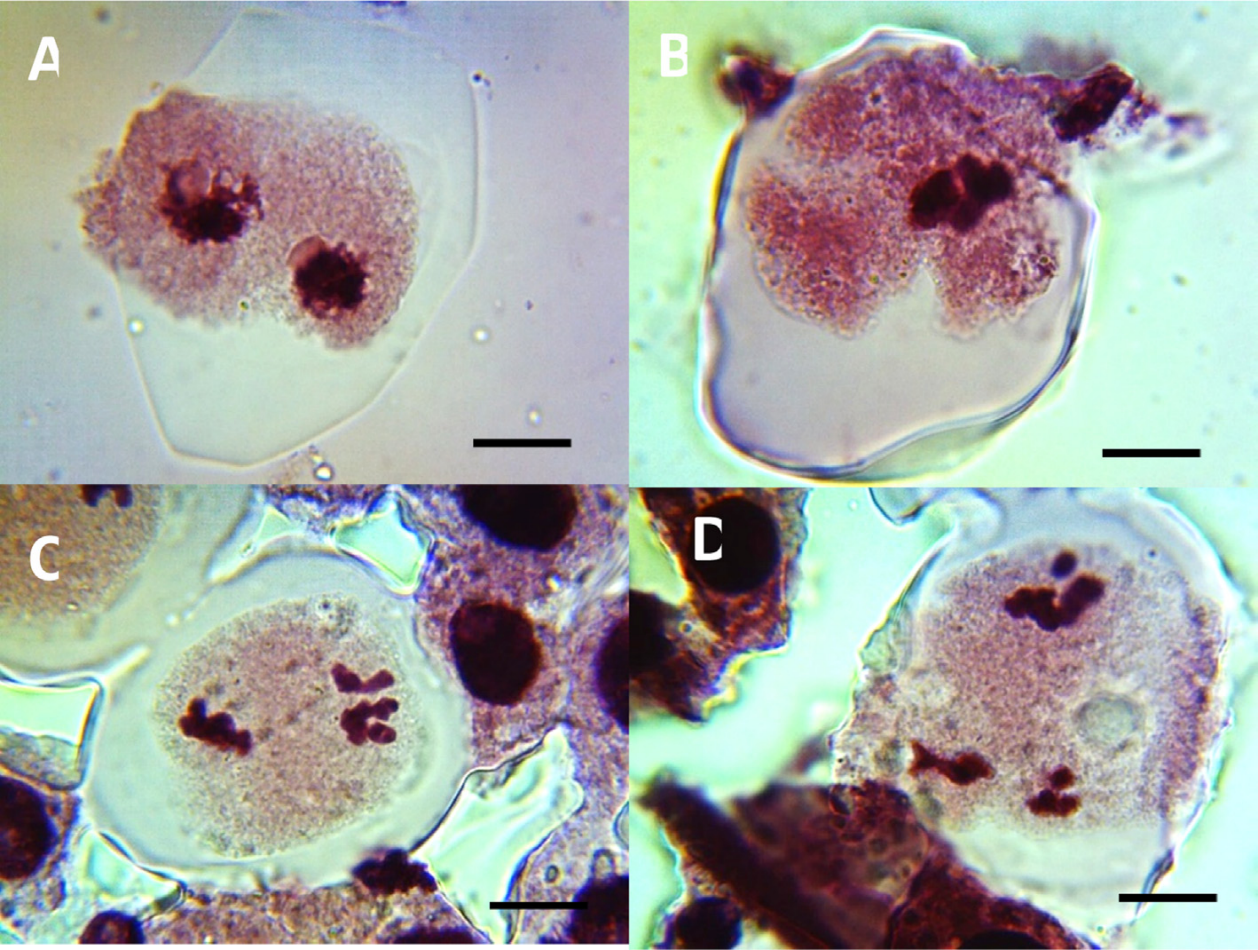
Syncytes induced by ME, IM and CL in PMCs of *P. sativum*. A. Syncytes in PI in PMCs, B. Syncytes in MI in PMCs, C. Syncytes in AI in PMCs, D. Syncytes in TI in PMCs of *P. sativum.* PI = Prophase I; MI = Metaphase I; AI = Anaphase I; TI = Telophase I; Scale bars = 10 μm.

In control group, no cytomixis cells were observed in various stages of meiosis I (PI, MI, AI and TI) [Table 3, Table 4 and Fig. 6 (A, B, C and D)]. At 0.1% ME treated seeds for 1 h, significant increase (p < 0.05) in syncyte cells was observed in PI phase which was (0.66 ± 0.02) and further increase in concentration from 0.2 to 0.5%, very significant increase (p < 0.01) in syncyte cells were noticed when compared to control. However, in MI, AI, TI, stages at 0.1%, a non-significant increase in syncyte cells in PMCs was observed and a significant increase (in 0.2 %- p < 0.05; 0.3 to 0.5%- p < 0.01) in syncyte cells in PMCs were observed at 1 h, when compared to control.

In case of IB treated seeds at 0.1% concentration for 1 h, significant increase (p < 0.01) in syncyte cells were noticed in PI and TI stage of meiosis I in PMCs. However, in MI and AI stage of meiosis I at 0.1% concentration, a non-significant increase in syncyte cells in PMCs was observed. From 0.2 to 0.5%, significant increase (p < 0.01) in syncyte cells were reported when compared to control at 1 h in various stages of meiosis I (PI, MI, AI and TI). In case of CL treated seeds at 0.1% concentration in PI stage of meiosis I at 1 h, a non-significant increase in syncyte cells was noticed and at 0.2 to 0.5% concentration, significant increase (p < 0.01) in syncyte cells were reported when compared to control. Further, in MI, AI, and TI stages of meiosis I at 1 h, significant increase (p < 0.01) in syncyte cells was observed from 0.1 to 0.5% when compared to control.

At 0.1% ME treated seeds, non-significant increase in syncyte cells were noticed in PI, MI, and TI phase, which was 0.33, 0.48 and 0.64% respectively and in AI phase, significant increase (p < 0.05) in syncyte cells (0.71%) was observed and further increase in concentrations from 0.2 to 0.5%, highly significant increase (p < 0.01) in syncyte cells were noticed when compared to control at 3 h. In case of IM and CL treated seeds, significant increase (p < 0.01) in syncyte cells were reported in PI, MI, AI and TI stages of meiosis I in PMCS at all the concentrations in comparison to control for 3 h.

## 4 Discussion

The results of the current study conducted on *P. sativum* further confirms that ME, IM, and CL induce pollen fertility, cytomixis cells, and syncytes cells in PMCs. Pollen fertility has been shown to decrease as ME, IM, and CL concentrations rise. The lower percentage of pollen fertility is an indication of disturbance in the reproductive process and it appears to be the result of all the cumulative events which leads to cytogenetical abnormalities, that ultimately influenced the reproductive attainment of microsporogenesis (Kravets, 2013, Siddiqui and Alrumman, 2020a, Siddiqui and Alrumman, 2020b, Girjesh and Shefali, 2020).

Cytomixis, the mechanism of transference of chromatin material between PMCs, has a significant impact on meiotic process and post meiotic outcomes. The one-way movement of nutritive constituents and numerous organelles from active PMCs to feebler ones take place via cytomictic channels, which originate in preexisting plasmodesmata system (Mursalimov et al., 2021b, Rosselló et al., 2022). Across cytoplasmic linkages and cytomictic pathways and by dissolution of cell wall, chromatin matter/chromosomes migrate amongst the adjacent PMCs (Mursalimov et al., 2016, Aksic et al., 2016, Mursalimov et al., 2021a). Cytomixis via cytoplasmic channels was found to be more common (Kravets, 2018, Rosselló et al., 2022). The nucleus and nuclear matter were transferred across single channels and multiple cytomictic channels at the same time. Although most of the cytomictic channels were found during early prophase, but by end of meiosis I, they have been closed by callose. A number of other researchers have made similar observations (Kumar and Singh, 2020, Ascari et al., 2020, Mursalimov et al., 2021a, Mursalimov et al., 2021b).

Numerous researchers recommended that cytomixis had a discrete influence on microsporogenesis, because movement of fragments or entire nucleus amongst generative cells via cytomictic channels might result in polyploid and aneuploid gametes. By a procedure termed as sexual polyploidisation (Veilleux, 1985), unreduced gametes create entities having higher ploidy levels and it is deemed to be the key route for polyploids formation (Kim et al., 2009). Cytomictic transmigration takes place when the cell walls of adjoining PMCs dissolve, resulting in the creation of syncytes (Kaur and Himshikha, 2017, Girjesh and Shefali, 2020).

The donor PMC chromatin substance was decreased and pulled near the place of cytomictic contact during the cytomixis process and subsequently transported to recipient cell via cytomictic channels (Mursalimov et al., 2021a). Such chromatin substances were removed in form of pyknotic chromosomes in accordance with the findings (Singhal and Kumar, 2008, Ascari et al., 2020). Abnormalities might be related to creation of genetically unbalanced cells, which leads to cell deterioration and sterile pollen grains (Girjesh and Shefali, 2020; Rosselló et al., 2022). Syncytes appear as a result of cytomictic transmigration amongst adjacent PMCs due to dissolution of cell wall (Harshita and Girjesh, 2018, Girjesh and Shefali, 2020, Mursalimov et al., 2021a).

In several cases, whole nucleus migration resulted in syncytes which generates unreduced pollen, resulting in the formation of polyploids. Syncyte appearance has been observed in the Poaceae (Boldrini et al., 2006), Fabaceae (Sarbhoy, 1980, Girjesh and Chaudhary, 2016) and Asteraceae (Kim et al., 2009), suggesting that it could be a normal mechanism in angiosperms. In the commencement of low-level polyploidy, formation of syncytes in diploid entities is critical and it has a major function in the formation of infraspecific polyploids (Bellucci et al., 2003, Kim et al., 2009).

Syncytes formation in the course of microsporogenesis has been found earlier in *Phleum pretense* (Levan, 1941), *Cyamopsis tetragonoloba* (Sarbhoy, 1980), Zea mays (Caetano Pereira et al., 1999), *Brachiaria decumbens* (Mendes Bonato et al., 2001), intergeneric hybrids of Triticeae such as *Psathyrostachys huashanica*x, *Secale montanum* (Wang, 1988) and *Roegneria ciliaris* × *Psathyrostachys huash* (Yen et al., 1993).

Certain structural changes might occur in PMCs of *P*. *sativum* under the influence of pesticides (Sengupta, and Sengupta, 2022). Free radicals have been found to cause genomic instability in cells. Reactive oxygen species are very unstable and can disrupt the cytoskeleton, induce energy metabolism imbalances, and damage DNA, resulting in chromosomal abnormalities (Gogoi et al., 2021, Ozel et al., 2022, Acar et al., 2022). Several investigations have shown that these pesticides change the redox status of plant cells in the past, (Bianchi et al., 2016, Acar et al., 2022, Kalefetoglu, 2021, Gogoi et al., 2021). Might be ME, IM, and CL are the contributory agents in this study, causing instigation of cytomixis and the generation of syncytes and gametes with changed number of chromosomes that can be used to improve some distinctive traits in plants.

## 5 Conclusion

The current study clearly demonstrates that induced cytomixis, syncytes and pollen fertility in PMCs generated by ME, IM, and CL treatments on *P. sativum* might be considered a likely source of polyploid gamete generation via manifestation of syncytes. The present study clearly demonstrates the genetic alterations caused by the pesticides. However, these gametes might be utilized in breeding operations to establish genetic diversity by altering the number of chromosomes.

## Declaration of Competing Interest

The authors declare that they have no known competing financial interests or personal relationships that could have appeared to influence the work reported in this paper.
